# A cost-reducing reimbursement programme? Effects of value-based reimbursement on healthcare costs

**DOI:** 10.3389/fpubh.2024.1326067

**Published:** 2024-12-11

**Authors:** Thérèse Eriksson, Hans Tropp, Ann-Britt Wiréhn, Lars-Åke Levin

**Affiliations:** ^1^Department of Health, Medicine and Caring Sciences (HMV), Linköping University, Linköping, Sweden; ^2^Department of Biomedical and Clinical Sciences, Linköping University, Linköping, Sweden; ^3^Center for Medical Image Science and Visualization, Linköping University, Linköping, Sweden; ^4^Unit for strategic healthcare in Region Östergötland, Linköping, Sweden

**Keywords:** value-based, reimbursement, bundled payment, pay-for-performance, PROM (patient reported outcome measures), cost, healthcare cost and resource utilization, EQ-5D

## Abstract

Value-based reimbursement programmes have become increasingly common in attempts to bend the cost curve of healthcare without negative effects on quality. The aim of this study was to analyse the effect of introducing a value-based reimbursement programme on the cost to third-party payer. We performed a retrospective observational study with a before and after design based on the introduction of a value-based reimbursement programme in Sweden. We analysed patient level cost data from inpatient and outpatient care of patients undergoing lumbar spine surgery, 2006–2015. The average 1-year episode cost decreased 11 percent during the first 2 years with the value-based reimbursement. The number of patients increased 22 percent during the same period, causing the total cost to increase by 8 percent. The value to third-party payer increased after the introduction of the value-based reimbursement since more patients were treated and attained a positive outcome. The decreased episode cost may be a result of better coordinated post-discharge care. Another explanation could be that costs previously borne by the third-party payer are shifted onto the healthcare providers. Thus, it is crucial that providers find a sustainable way of delivering care in the long term to retain value. Interlinking patient records facilitates a holistic perspective among healthcare providers raising awareness of health care utilization through the whole care chain.

## 1 Introduction

To promote quality in healthcare and at the same time bend the cost curve, reimbursement has shifted from fee-for-service to alternative payment models, such as value-based reimbursement. Value-based reimbursement programmes (VBRP) strives to synchronize financial incentives with professional values, often by integrating multiple payment models, e.g., bundled payment and pay-for-performance (P4P) (1). Other commonly used terms for VBRP include value-based payment models (2) and value-based purchasing models (3). Ideally, a VBRP promotes both quality improvement and cost control to deliver value defined as “the health outcomes achieved that matter to patients relative to the cost of achieving those outcomes” (4). Consequently, value is conceptualized as the ratio between outcome and cost (5). In theory, a VBRP contains both quality enhancing and cost-containing incentives to generate value (4, 6).

In this study, we analyse how the cost to third-party payer (Region Stockholm) is affected by the introduction of a VBRP within elective spine surgery in Region Stockholm, Sweden. Low back pain affects an estimated 80–85 percent of the global population at some point in their life (7), resulting in a significant and growing economic burden (8) where indirect costs has been estimated to account for 66–84 percent of the total cost (9, 10). Thus, an effective reimbursement programme that considers the full care chain is essential. A VBRP with incentives for a holistic approach may be particularly suited for spine surgery, given the variability in clinical guidelines and the incomplete evidence regarding who benefits from surgical intervention (11). The Stockholm VBRP (STHLM-VBRP) has a unique design because it extends the financial responsibility of healthcare providers to include all healthcare utilization related to spine surgery for 1 year following the index surgery, including treatment of complications and physiotherapy. As a result, providers face a more extensive financial responsibility than those in other assessed programmes (12, 13). Further, the performance measure used in the STHLM-VBRP is based on the level of pain the patient reports 1 year after surgery.

Systematic literature reviews on VBRP (1, 3, 14–16) provides mixed evidence on the effect on cost, as well as reviews on the specific features included in the STHLM-VBRP such as P4P (17–20), and bundled payment (21–23). Later systematic reviews of VBRP however have shown promising results in terms of lower spending growth with equal or improved quality (15). One study has so far shown that the STHLM-VBRP had no negative effect on quality (24). However, to assess whether the STHLM-VBRP increased value or not, the cost-implications needs to be investigated.

The overall aim of this study is to analyse the association between costs to third-party payer (Region Stockholm) and the introduction of a value-based reimbursement programme (STHLM-VBRP).

## 2 Materials and methods

### 2.1 Healthcare setting

Sweden has universal health coverage and the healthcare system is publicly financed. There are 21 self-governing regions in Sweden with the responsibility to provide and finance healthcare through tax-revenues. Region Stockholm accounts for 26 percent of the inpatient care in Sweden (25) and is thereby the largest region.

For a private healthcare provider to deliver care, they must establish a commissioning contract with the relevant region. A commissioning contract is established either through the Public Procurement Act (26) or through the Act on Systems of Choice (27). Within the healthcare context, the Act on Systems of Choice is more commonly known as Patient Choice, which is the term we will use hereafter. Under the Public Procurement Act, each healthcare provider specifies the price at which they can perform a certain number of surgeries. If the Region accept this price, the provider is authorized to carry out the agreed-upon number of surgeries for the contracted period. Within the framework of Patient Choice, the Region sets the price level that healthcare providers can opt to accept, without any restriction on volume. The idea of Patient Choice is that the preferences of patients should drive competition. This approach emphasizes competition based on quality rather than price, which is essential for value-based healthcare (28).

In 2013, Region Stockholm switched from contracting healthcare providers through the Public Procurement Act to Patient Choice in elective spine surgery (i.e., surgeries that are scheduled in advance and does not involve any emergency). It was further decided that a value-based reimbursement programme (VBRP) should be introduced simultaneously. Both public and private healthcare providers perform elective spine surgery in Region Stockholm. However, Patient Choice and the VBRP only include the private healthcare providers. There were three accredited private healthcare providers at the time of the introduction of Patient Choice and they performed most of the surgeries. The STHLM-VBRP encompasses both degenerative lumbar and cervical surgeries; however, not all healthcare providers were accredited for cervical spine surgery. Consequently, this study focuses on degenerative lumbar surgery. The categories are defined by specific diagnoses and surgical procedures (see Supplementary Table S1) and have been employed for an extended period in the national quality registry for spine surgery (SweSpine) (29).

### 2.2 The value-based reimbursement programme

An extensive description of the Stockholm VBRP (STHLM-VBRP) can be found in previous published studies (24, 30, 31) but the main features are summarised below.

**Figure 1:**
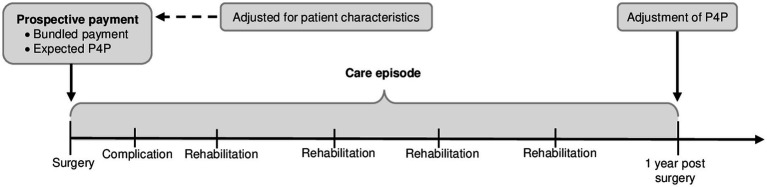
Illustration of the value-based reimbursement programme used in elective spine surgery in Region Stockholm (STHLM-VBRP), Sweden. Modified illustration from Eriksson et al. (24).

The healthcare provider receives a prospective payment when the surgical procedure is registered. This payment comprises a bundled payment and an expected performance-based payment (Figure 1). The bundled payment should cover the costs of the spine surgery but also care related to the surgery during the following year. Hence, the healthcare provider receives no further payment to treat complications, reoperations and rehabilitation visits related to the surgery. When surgically treated patients seek care elsewhere, the healthcare provider that received the bundled payment will be billed. Thus, to stimulate an effective and integrated care chain, the cost responsibility is extended to include healthcare that is supplied by other healthcare providers. The complication and rehabilitation activities depicted in Figure 1 are just examples of activities that were previously reimbursed separately.

To promote needs-based healthcare, it is essential to minimize financial risk disparities among patients. The STHLM-VBRP limits the financial risk between patients by adjusting the prospective payment for age, gender, and comorbidity level. Further, surgical procedures that involve surgery on more than two levels of the spine generate an additional payment to the provider.

The performance-based payment is used as a complement to the bundled payment to promote a focus on quality but also to prevent healthcare provider from skimping on necessary care. The performance-based payment used in STHLM-VBRP is based on the outcome measure Global Assessment (GA), which involves a retrospective transition question asked 1 year after surgery (“How is your back/leg pain today compared to before the surgery?”). The performance-based payment is based on leg pain for patients with disc herniation or spinal stenosis, and back pain for patients with segmental dysfunction and spondylolisthesis. The patient can choose between six response options (pain free, much better, somewhat better, unchanged, worse, did not have pain before the surgery) (32). The registration of GA is administered and managed by SweSpine (33). Importantly, healthcare providers are not in any way involved in this process.

The expected P4P, which is included in the prospective payment to healthcare providers, is based on national historic outcomes of GA registered in SweSpine. One year after surgery, the expected P4P is adjusted according to the actual patient reported outcome of GA. Patients with better results than predicted generated a positive adjustment in the range of 1 to 6 percent of the prospective payment (24). Whereas patients with worse results than predicted generated a negative adjustment in the range of −1 to −18 percent of the prospective payment (24). Thus, there are stronger financial incentives associated with avoiding negative outcomes compared with reaching positive outcomes.

### 2.3 Study population

The study population consisted of patients ≥18 years of age, living in Region Stockholm and undergoing lumbar spine surgery 2006–2015. Patients were included based on diagnosis (ICD-10) and surgical procedure code (NCSP). The new reimbursement programme only includes private healthcare providers. Hence, only patients who were surgically treated by a private healthcare provider were included in the analysis.

### 2.4 Study design and data sources

This was a retrospective observational register study, using a before and after design. The value-based reimbursement programme was introduced in October 2013; hence the study period contains 7.75 years before the introduction and 2.25 years after the introduction. Data were collected until the end of 2016 to account for the reimbursement adjustment 1 year after surgery for patients surgically treated in 2015.

Patient level data on diagnosis, surgical procedure, age, gender, costs, were extracted from the Stockholm regional patient registry (VAL). When a patient was identified with the right combination of diagnosis and surgical procedure code for spine surgery, all healthcare utilization was extracted for the following year. Socioeconomic data on income, educational level, employment, and country of birth were extracted from Statistics Sweden. Patient reported outcome measures were extracted from Swespine.

The National Board of Health and Welfare anonymised and interlinked data from VAL, Swespine and Statistics Sweden. Data were obtained with ethical approval.

### 2.5 Study variables

The primary outcome was total and mean episode cost for patients undergoing lumbar spine surgery, from the perspective of a third-party payer, which in our case was Region Stockholm. The costs included were all healthcare costs during the 1-year episode after lumbar spine surgery, which includes inpatient care, outpatient care, primary care, and rehabilitation.

To compare the populations undergoing surgery before and after the introduction of the STHLM-VBRP we controlled for patient baseline characteristics by including variables on age, gender, body mass index (BMI), comorbidity level measured with Charlson comorbidity index (34), EQ-5D-3L, Oswestry Disability Index (ODI), annual income, educational level, employment status, and whether the patient was born outside of Europe. EQ-5D-3L comprises five dimensions (mobility, self-care, usual activities, pain/discomfort and anxiety/depression), with three levels (no problems, some problems, and extreme problems) (35). In Swespine the EQ-5D-3L has been converted into a single summary index using the tariff by Dolan (36). The ODI is a recommended condition-specific outcome measures for spinal disorders (37, 38) and comprises ten items: pain intensity, personal care, lifting, walking, sitting, standing, sleeping, sex life, social life, and traveling. Based on these items, patients get a percentage score of disability, where 0 percent corresponds to no disability and 100 percent corresponds to full disability.

### 2.6 Analysis

Patient characteristics are described using proportions for categorical data and mean and standard deviation for continuous data, differences between populations was tested using student’s *t*-test and Wilcoxon rank sum test for categorical variables. Analysis of patient characteristics of the same patient population has previously been published in Eriksson et al. (24).

To assess the effect of the STHLM-VBRP on cost to the third-party payer (Region Stockholm), we analysed the total episode cost per year and the mean episode cost of patients surgically treated from 2006 until the end of 2015. The STHLM-VBRP contract holds healthcare providers financially responsible for related services (complication treatment and physiotherapy) for 1 year following surgery. In our data however, we are not able to distinguish related healthcare from unrelated. Therefore, we include all healthcare utilization in the year following surgery, assuming that unrelated healthcare remains consistent before and after the introduction of STHLM-VBRP. Thus, the episode cost encompasses not only the cost of spine surgery but also all healthcare utilization – including primary, outpatient and inpatient care – during the year following the index surgery.

As a first step, costs are presented comparing the two last years prior to the introduction of the STHLM-VBRP with the two first years following the introduction. The second step takes a longer time-perspective into account to compare trend and level 2006–2015. We used segmented regression analysis (39) to assess potential changes in level and trend of costs over time, from 2006 until the end of 2015. The time series (2006–2015) is interrupted by the introduction of STHLM-VBRP in 2013, creating two segments of interest. This allows us to identify changes in trend and level of costs after the introduction. The independent variables used were: *Time*, indicating the number of months after January 2006; *VBRP*, indicating the introduction of the STHLM-VBRP in 2013; *Time after*, indicating the number of months after the introduction of the STHLM-VBRP; *July*, indicating the month of July since fewer patients undergo surgery during this month due to summer holidays. A sensitivity analysis was conducted based on a shorter period (2011–2015) but with equal amount of data points pre- and post-intervention.

Patients with missing values in reimbursement were excluded from the analysis. Statistical significance was assessed at the 5 percent level. Analyses were performed using SAS 9.4. All costs were adjusted to the 2016 price level and presented in EUR with an exchange rate corresponding to 1 SEK = 0.11 EUR.

## 3 Results

From year 2006 until the end of 2015 were 10,389 patients surgically treated for lower back pain; 6,738 patients were treated before the introduction of the VBRP and 3,651 after the introduction. There were no significant differences between patients surgically treated after the introduction compared to before the introduction of STHLM-VBRP regarding age, gender, BMI, EQ-5D level prior to surgery and educational level. The comorbidity level of patients treated after the introduction (M = 0.31, SD = 0.78) was higher (*p* < 0.01) compared to the comorbidity level of patients treated before the introduction (M = 0.24, SD = 0.71), indicating a slightly sicker patient group after the introduction. However, the ODI-level indicated a less impaired population (*p* = 0.16) after the introduction (M = 41.16, SD = 16.41) compared to before the introduction (M = 41.88, SD = 15.87). Patients treated after the introduction had a higher (*p* < 0.01) annual income (M = € 31,185, SD = € 44) compared to patients treated before the introduction (M = € 27,449, SD = 26,053). Further, the employment rate among patients treated after the introduction (M = 54.73, SD = 49.78) was higher (*p* = 0.94) compared to patients treated before the introduction (M = 52.67, SD = 49.93). An increased annual income and a higher employment rate indicate higher socioeconomic status among patients surgically treated after the introduction of the VBRP. However, the proportion of patients born outside of Europe was higher (*p* < 0.01) after the introduction (M = 12.01, SD = 29.34) compared to before the introduction (M = 8.22, SD = 22.47).

**Figure 2:**
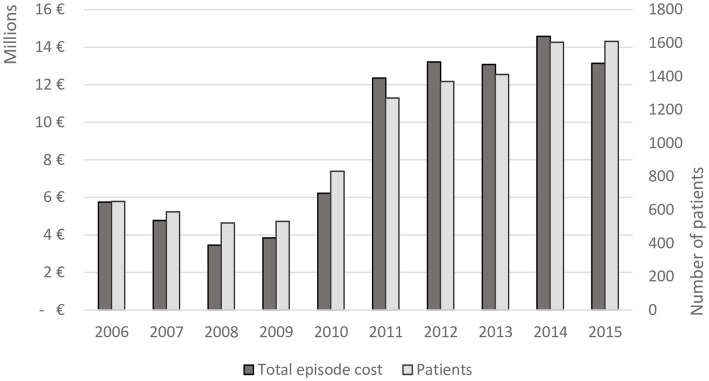
The total episode cost and number of surgically treated patients for the time period 2006–2015 in Region Stockholm.

The number of patients surgically treated by private healthcare providers increased during the study period. The total annual episode cost did also increase with the exception of 2013 and 2015. Figure 2 depicts the development for the total annual episode cost and number of surgically treated patients each year for the period 2006–2015. Comparing the last 2 years before the introduction of the STHLM-VBRP (2011–2012) with the two first years after the introduction (2014–2015), the number of surgically treated patients increased by 22 percent, from 2,641 to 3,215 patients. The total aggregated episode cost of surgically treated patients during the first 2 years with the VBRP, i.e., 2014–2015, amounted to € 27.7 million, which is an 8 percent increase compared with the two last years before the introduction, i.e., 2011–2012. The mean episode cost per surgically treated patient was 11 percent lower during the two first years after the introduction of the VBRP (2014–2015) compared with the two last years before the introduction (2011–2012), € 8,620 compared to € 9,682, respectively.

**Figure 3:**
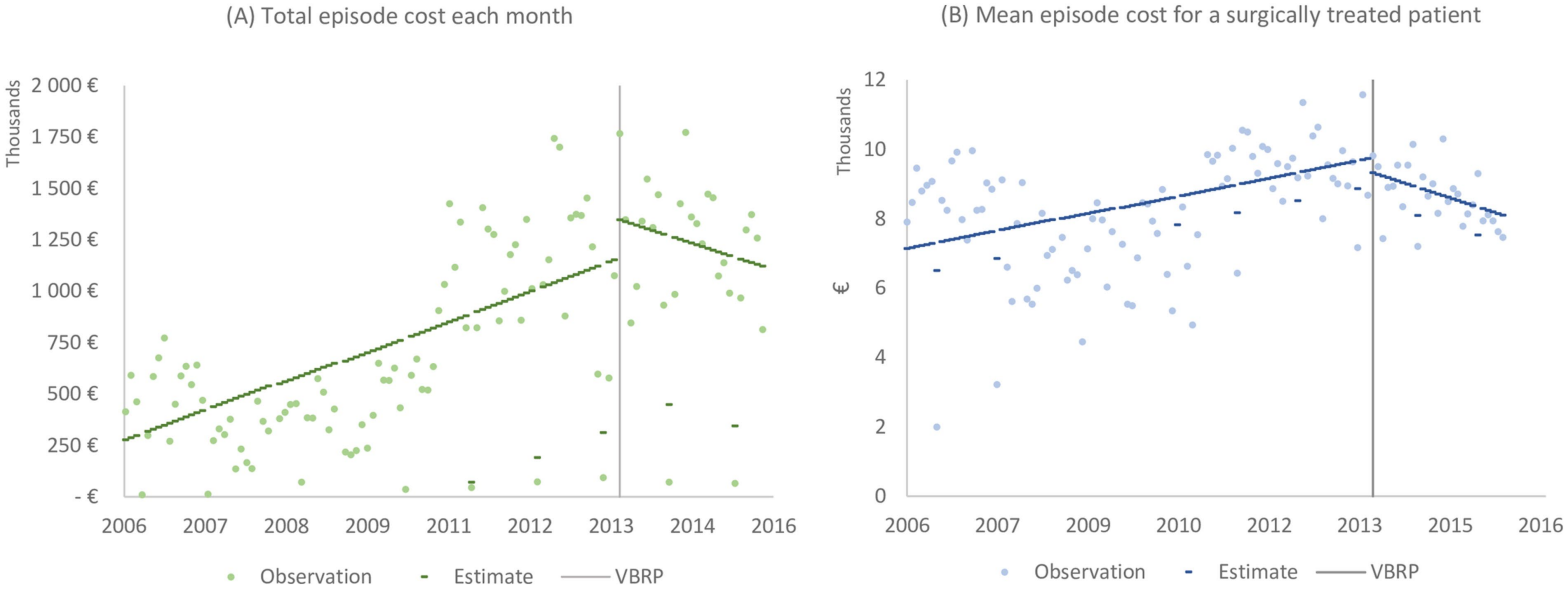
The total (A) and mean (B) episode cost for surgically treated patients. The vertical line indicates the introduction of the STHLM-VBRP at the end of 2013.

The result of the segmented regression analysis when considering a longer time frame (i.e., 2006–2015) is presented in Table 1. The total episode cost for all patients surgically treated in January 2006 was estimated at € 236,862. In total, the cost for all surgically treated patients in 2006 reached € 5.7 million. The *Time* parameter in Table 1A represents the trend prior to the introduction of the VBRP, indicating that the total episode cost increased by €10,051 (*p* < 0.0001) each month from January 2006. However, following the introduction of the VBRP, the trend shifted to negative (represented by the parameter *Time* after), counteracting the previous positive trend (see Figure 3A). Consequently, the total episode cost decreased by € 8,684 (*p* = 0.0103, € 10,051 – € 18,735) each month after the introduction of the VBRP. The total episode cost of patients surgically treated in 2015 amounted to € 13.1 million. Following the introduction of the VBRP, the total episode cost level increased with €204,010, however not statistically significant (*p* = 0.1146).

**Table 1:** Parameter estimates predicting the total and mean episode cost of surgically treated patients, 2006–2015.

	(A) Total episode cost per month (€)			(B) Mean episode cost (€)		
Parameter	Estimate	SE	*p*-value	Estimate	SE	*p*-value
Intercept (Jan 2006)	236,862	60,758	0.0002	7,104	408	<0.0001
Time	10,051	1,146	<0.0001	29	8	0.0003
VBRP (Oct 2013)	204,010	128,300	0.1146	−360	861	0.6763
Time after	−18,735	7,178	0.0103	−76	48	0.1185
July	−820,354	105,189	<0.0001	−808	706	0.2547

SE, standard error; Intercept, cost level in January 2006; Time, number of months from January 2006; VBRP, indicates the introduction of the STHLM-VBRP at the end of 2013, which is 92 months after January 2006 (Time = 92); Time after, number of months after the introduction of VBRP (hence Time-91); July, indicates the month of July.

Table 1B presents the estimates for level and trend of the mean episode cost of a surgically treated patient between 2006 and 2015. The mean episode cost of a patient surgically treated in January 2006 was estimated at € 7,104 (<0.0001). Each month, the annual cost per patient increased by € 29 (*p* = 0.0003). The introduction of the VBRP reduced the level of the mean cost per patient by € 360 (*p* = 0.6763) and the trend by € 47 per month (*p* = 0.1185, € 29 before VBRP – € 76 after VBRP), however these changes are not statistically significant. The observations and estimated mean episode cost per patient is depicted in Figure 3B.

A sensitivity analysis conducted over a shorter period (2011–2015), using 27 data points both before and after the intervention, reveals a similar negative trend in costs, although it is not statistically significant (see Supplementary Table S2; Supplementary Figure S1).

## 4 Discussion

In this study we analysed the effect of the STHLM-VBRP on cost to a third-party payer (Region Stockholm). Our results show that the mean episode cost for surgically treated patients decreased by 11 percent following the introduction of STHLM-VBRP. However, due to a 22 percent increase in the number of surgically treated patients, the total episode cost for spine surgery increased by 8 percent. Thus, the introduction of STHLM-VBRP is associated with decreasing costs while, as reported by Eriksson et al. (24), maintaining patient reported outcome measures (PROM) and thereby enhancing value as defined by Porter (4).

The lower mean cost may result from better coordinated care due to the increased financial responsibility of healthcare providers for post-discharge care, helping to avoiding unnecessary treatments. However, this reduced cost to Region Stockholm could come at the expense of the private healthcare providers. A fourth healthcare provider received accreditation in 2017 (31), suggesting that the reimbursement level was sufficient to attract new healthcare providers. Nevertheless, one of the original healthcare providers closed its business in 2021 due to competitive pressure and strained economy (40). Understanding how this shift in market structure has impacted costs and PROMs would be valuable for assessing long-term consequences of the STHLM-VBRP. When costs are shifted to private healthcare providers concerns arise about whether patients continue to receive adequate care after surgery. Previous studies on the population covered by the STHLM-VBRP found no evidence for negative effects on PROM following the introduction of the VBRP (24).

Consistent with our findings, a multicentre observational study of patients undergoing lumbar fusion reported a reduction in costs following introduction of bundled payment (41). In contrast, Bronson et al. (42), found no effect on cost after introducing bundled payment in spine surgery, while Jubelt et al. (43) reported an increase in costs. Both studies observed a more complex patient mix after the introduction of the programme. Differences in design and context may further explain the heterogenous results, including a shorter episode duration of 90 days compared to the 365-day episode in STHLM-VBRP, and the fact that bundled payment was not combined with P4P. Our study is based on patients surgically treated by publicly financed private healthcare providers in Sweden, whereas the studies by Bronson et al. (42) and Jubelt et al. (43) were conducted at hospital and university clinics in the U.S., respectively. Our study is so far the only one to assess the effect of a one-year bundled payment episode combined with pay-for-performance (P4P) using PROM.

Overall, our results align with studies demonstrating promising outcomes in terms of reduced spending growth and sustained quality levels (15, 17, 23, 44). While systematic reviews have historically presented mixed evidence regarding Value-Based Reimbursement Programmes (VBRPs) (17, 45, 46), the generalisability of these findings has been limited by variation in the design of reimbursement programmes and the context of their implementation, but also by methods used for assessment (3, 45). In response to these limitations, an increasing number of studies have developed frameworks to identify both facilitating and inhibiting factors for a successful VBRPs (2, 3, 6, 15, 16, 47). A key factor often overlooked in these frameworks, as highlighted in this study, is the significance of time, which is crucial for a thorough assessment of the long-term effects of VBRPs.

In the study by Eriksson et al. it was reported that the P4P within STHLM-VBRP was a “stick” rather than a “carrot” for health care providers (24). This in combination with a financial responsibility for a year makes it crucial that healthcare providers come to an understanding of which patients benefit from a surgery and which patients do not. This is something which is continually debated within spine surgery (30, 31). A previous study indicates that socioeconomic factors (education, income, unemployment and country of birth) also affect the outcome of spine surgery (48). In other studies (42, 43), authors argued that the existing DRG system is not well-suited to manage the increasing case complexity and heterogeneity present in modern spinal surgery. It has further been argued that value-based reimbursement in collectively-financed healthcare requires monitoring of socioeconomic patient data in order to maintain equality in service provision (49). Hence, it would probably be beneficial to include risk adjustment of reimbursement based on both clinical and socioeconomic variables to avoid discrimination (48). In the case of the STHLM-VBRP, each payment was individually risk adjusted and the traditional use of DRG was replaced with new categories that were already established in SWESPINE. However, there is no adjustment based on socioeconomic factors. The higher socioeconomic status of patients receiving surgical treatment after the introduction of VBRP may suggest a potential case of cherry-picking. However, the increase in the proportion of patients born outside of Europe after the introduction of VBRP counters this notion, as this demographic factor is considered a risk factor in surgical outcomes in Sweden (48). Another possible explanation for the shift in patient characteristics is the removal of annual limits on the number of patients each healthcare provider could treat. This enables healthcare providers to treat a larger number of patients, potentially leading to a more diverse patient population. Customised reimbursement based on patient-level data may lead to better distribution of healthcare resources towards needs-based healthcare. To reduce incentives for “cherry-picking,” a future update of the STHLM-VBRP should include adjustments based on socioeconomic variables.

A limitation of our study is that our dataset does not include patients that were referred to a spine surgeon for assessment but then not surgically treated. Therefor we cannot assess whether cherry-picking or a shift in indications occurred in that part of the care chain. Since the indications for surgery can be rather vague within elective spine surgery there is a risk of “over-treating” patients. This may result in negative side-effects both financially and medically since surgical procedures are more costly and have a greater risk of adverse events. Further, some surgical procedures only show modestly better effect than conservative treatment (11, 50). A concern when providers have no restriction regarding volume is that it might lead to an increased procedural volume of spine surgery without regard to quality, and thus drive cost and diminish the value of spine care (51, 52). To weed out providers that deliver low value care, those who deliver superior outcomes must be rewarded (38). The STHLM-VBRP reward healthcare providers that were able improve the pain of their patients (24), however, when the pain was unchanged or worse healthcare provider had to repay money to Region Stockholm. Thus, the STHLM-VBRP may weed out providers that deliver low value care and increase value to third party payer by decreasing the costs.

Another limitation is due to the observational approach of our study, using a natural experiment design we can only test for association and not causality. Our method introduces various biases; one strategy to minimize confounding and regression to the mean was to extract data over an extended period (i.e., 2006–2015). Using segmented regression analysis allows us to compare the slopes over time between pre-intervention and post-intervention periods and to assess any outcome discontinuity (change in level) that could occur when the intervention began (58). It further allows us to adjust for seasonal trends, e.g., the month of July was included as a parameter in the regression analysis because of holiday season, during which very few patients undergo surgery, thereby impacting the total cost of spine surgery for that month.

A third limitation is that our data only cover the first 2 years (or 27 moths) with the new reimbursement programme. Our sensitivity analysis indicated that 27 months is insufficient to establish a negative association between VBRP and costs. Previous research by Song et al. (53) has shown that larger improvements in quality do not occur immediately when introducing a VBRP. Thus, it takes time for providers to adapt to the structures of a new reimbursement programme (54) and it is common with transition periods during the implementation (55) that are characterised with “child diseases” and may cause a drop in quality of care (56). Thus, a “wash-out” period would be appropriate to remove potential transition effects. Nevertheless, the transition is an inevitable part of the introduction of a new reimbursement programme and important to consider when assessing the effects since it reflects the first 2 years of using a VBRP.

Lastly, our results reflect the costs from the perspective of a third-party payer, which in our case is Region Stockholm. Consequently, we do not know whether the cost for healthcare providers decreased, therefore we do not know how sustainable this reimbursement programme is. Investigating the profitability of private healthcare providers under the VBRP would be valuable for assessing the true cost of spine surgery. Have the private healthcare providers succeeded in making post-surgery more effective or do they struggle to make ends meet? Do they have a chance to adapt to the new reimbursement level given the load of post-discharge care? Other important aspects affecting the future viability of the STHLM-VBRP is the administrative burden and the micropolitical and professional issues that are in play (57). Only time will provide answers to these questions. As will time show whether these healthcare providers manage to treat these patients efficiently without any negative impact on quality.

## 5 Conclusion

The cost per patient to third-party payer decreased when a value-based reimbursement programme was used for elective spine surgery. Due to the decreased cost per patient without negative effects on quality or access to care, the STHLM-VBRP seems promising. In order to hold healthcare providers responsible for post-discharge care it is necessary to interlink data from patient registries when using bundled payment. Interlinking patient records facilitates a holistic perspective among healthcare providers raising awareness of healthcare utilization throughout the care chain.

## Data Availability

The raw data supporting the conclusions of this article will be made available by the authors, without undue reservation.

## References

[1] Damberg CL Sorbero ME Lovejoy SL Martsolf GR Raaen L Mandel D. Measuring success in health care value-based purchasing programs: findings from an environmental scan, literature review, and expert panel discussions. Rand Health Q. (2014) 4:9. PMID: 28083347 10.7249/rhq-04-03-09 PMC5161317

[2] Cattel D Eijkenaar F Schut FT. Value-based provider payment: towards a theoretically preferred design. Health Econ Policy Law. (2020) 15:94–112. doi: 10.1017/S1744133118000397, PMID: 30259825

[3] Pandey A Eastman D Hsu H Kerrissey MJ Rosenthal MB Chien AT. Value-based purchasing design and effect: a systematic review and analysis. Health Aff (Millwood). (2023) 42:813–21. doi: 10.1377/hlthaff.2022.01455, PMID: 37276480 PMC11026120

[4] Porter ME. What is value in health care? N Engl J Med. (2010) 363:2477–81. doi: 10.1056/NEJMp1011024 21142528

[5] Porter ME Lee TH. The strategy that will fix health care. Harv Bus Rev. (2013) 91:50–70.23898735

[6] Conrad DA. The theory of value-based payment incentives and their application to health care. Health Serv Res. (2015) 50:2057–89. doi: 10.1111/1475-6773.12408, PMID: 26549041 PMC5338202

[7] Hoy D March L Brooks P Woolf A Blyth F Vos T. Measuring the global burden of low back pain. Best Pract Res Clin Rheumatol. (2010) 24:155–65. doi: 10.1016/j.berh.2009.11.002, PMID: 20227638

[8] Olafsson G Jonsson E Fritzell P Hagg O Borgstrom F. A health economic lifetime treatment pathway model for low back pain in Sweden. J Med Econ. (2017) 20:1281–9. doi: 10.1080/13696998.2017.1372252, PMID: 28840772

[9] Olafsson G Jonsson E Fritzell P Hägg O Borgström F. Cost of low back pain: results from a national register study in Sweden. Eur Spine J. (2018) 27:2875–81. doi: 10.1007/s00586-018-5742-6, PMID: 30155730

[10] Ekman M Johnell O Lidgren L. The economic cost of low back pain in Sweden in 2001. Acta Orthop. (2005) 76:275–84. doi: 10.1080/00016470510030698, PMID: 16097556

[11] Foster NE Anema JR Cherkin D Chou R Cohen SP Gross DP. Prevention and treatment of low back pain: evidence, challenges, and promising directions. Lancet. (2018) 391:2368–83. doi: 10.1016/S0140-6736(18)30489-6, PMID: 29573872

[12] Ryan AM. Medicare bundled payment programs for joint replacement: anatomy of a successful payment reform. JAMA. (2018) 320:877–9. doi: 10.1001/jama.2018.11787 30193258

[13] Ugiliweneza B Kong M Nosova K Huang KT Babu R Lad SP. Spinal surgery: variations in health care costs and implications for episode-based bundled payments. Spine. (2014) 39:1235–42. doi: 10.1097/BRS.0000000000000378 24831503

[14] Scott A Liu M Yong J. Financial incentives to encourage value-based health care. Med Care Res Rev. (2018) 75:3–32. doi: 10.1177/1077558716676594 27815451

[15] Cattel D Eijkenaar F. Value-based provider payment initiatives combining global payments with explicit quality incentives: a systematic review. Med Care Res Rev. (2020) 77:511–37. doi: 10.1177/1077558719856775, PMID: 31216945 PMC7536531

[16] Leao DLL Cremers HP van Veghel D Pavlova M Hafkamp FJ Groot WNJ. Facilitating and inhibiting factors in the design, implementation, and applicability of value-based payment models: a systematic literature review. Med Care Res Rev. (2023) 80:467–83. doi: 10.1177/10775587231160920, PMID: 36951451 PMC10469482

[17] Eijkenaar F Emmert M Scheppach M Schoffski O. Effects of pay for performance in health care: a systematic review of systematic reviews. Health Policy. (2013) 110:115–30. doi: 10.1016/j.healthpol.2013.01.008, PMID: 23380190

[18] Kondo KK Damberg CL Mendelson A Motu'apuaka M Freeman M O'Neil M. Implementation processes and pay for performance in healthcare: a systematic review. J Gen Intern Med. (2016) 31:61–9. doi: 10.1007/s11606-015-3567-0, PMID: 26951276 PMC4803682

[19] Christianson JB Leatherman S Sutherland K. Lessons from evaluations of purchaser pay-for-performance programs: a review of the evidence. Med Care Res Rev. (2008) 65:5s–35s. doi: 10.1177/1077558708324236, PMID: 19015377

[20] Mathes T Pieper D Morche J Polus S Jaschinski T Eikermann M. Pay for performance for hospitals. Cochrane Database Syst Rev. (2019) 7:Cd011156. doi: 10.1002/14651858.CD011156.pub2 31276606 PMC6611555

[21] Shih T Chen LM Nallamothu BK. Will bundled payments change health care? Examining the evidence thus far in cardiovascular care. Circulation. (2015) 131:2151–8. doi: 10.1161/CIRCULATIONAHA.114.010393, PMID: 26078370 PMC4471872

[22] Feldhaus I Mathauer I. Effects of mixed provider payment systems and aligned cost sharing practices on expenditure growth management, efficiency, and equity: a structured review of the literature. BMC Health Serv Res. (2018) 18:996. doi: 10.1186/s12913-018-3779-1, PMID: 30587185 PMC6307240

[23] Agarwal R Liao JM Gupta A Navathe AS. The impact of bundled payment on health care spending, utilization, and quality: a systematic review. Health Aff (Millwood). (2020) 39:50–7. doi: 10.1377/hlthaff.2019.00784, PMID: 31905061

[24] Eriksson T Tropp H Wiréhn A-B Levin L-Å. A pain relieving reimbursement program? Effects of a value-based reimbursement program on patient reported outcome measures. BMC Health Serv Res. (2020) 20:805. doi: 10.1186/s12913-020-05578-8, PMID: 32847579 PMC7450562

[25] The National Board of Health and Welfare. The national patient register The National Board of Health and Welfare (2020).

[26] SFS 2016:1145. The Public Procurement Act (LOU).

[27] SFS 2008:962. The Act on Systems of Choice (LOV).

[28] Porter ME. Value-based health care delivery. Ann Surg. (2008) 248:503–9. doi: 10.1097/SLA.0b013e31818a43af 18936561

[29] Stromqvist B Fritzell P Hagg O Jonsson B Sanden B. Swespine: the Swedish spine register: the 2012 report. Eur Spine J. (2013) 22:953–74. doi: 10.1007/s00586-013-2758-9, PMID: 23575657 PMC3631024

[30] Eriksson T Levin L Nedlund AC. Centrality and compatibility of institutional logics when introducing value-based reimbursement. J Health Organ Manag. (2021) 35:298–314. doi: 10.1108/JHOM-01-2021-0010, PMID: 34535988 PMC9136856

[31] Eriksson T Levin L Nedlund AC. The introduction of a value-based reimbursement programme-alignment and resistance among healthcare providers. Int J Health Plann Manag. (2023) 38:129–48. doi: 10.1002/hpm.3574, PMID: 36109866 PMC10087818

[32] Parai C Hagg O Lind B Brisby H. The value of patient global assessment in lumbar spine surgery: an evaluation based on more than 90,000 patients. Eur Spine J. (2018) 27:554–63. doi: 10.1007/s00586-017-5331-0 29058135

[33] Swedish Society of Spinal Surgeons. Swespine 25 years 2018 annual report. (2018). Report No.: 19. Available at: https://www.medscinet.com/swespine/uploads/swespine-arsrapport-english-2018.pdf

[34] Sundararajan V Henderson T Perry C Muggivan A Quan H Ghali WA. New ICD-10 version of the Charlson comorbidity index predicted in-hospital mortality. J Clin Epidemiol. (2004) 57:1288–94. doi: 10.1016/j.jclinepi.2004.03.012, PMID: 15617955

[35] EuroQol Research Foundation. EQ-5D-3L User Guide. (2018). Available at: https://euroqol.org/wp-content/uploads/2023/11/EQ-5D-3LUserguide-23-07.pdf

[36] Dolan P. Modeling valuations for EuroQol health states. Med Care. (1997) 35:1095–108. doi: 10.1097/00005650-199711000-00002 9366889

[37] Fairbank JC Pynsent PB. The Oswestry disability index. Spine. (2000) 25:2940–53. doi: 10.1097/00007632-200011150-00017 11074683

[38] Hills JM Weisenthal B Sivaganesan A Bydon M Archer KR Devin CJ. Value based spine care: paying for outcomes, not volume. Semin Spine Surg. (2019) 31:12–9. doi: 10.1053/j.semss.2018.07.004

[39] Wagner AK Soumerai SB Zhang F Ross-Degnan D. Segmented regression analysis of interrupted time series studies in medication use research. J Clin Pharm Ther. (2002) 27:299–309. doi: 10.1046/j.1365-2710.2002.00430.x 12174032

[40] Radio Sweden [Sveriges Radio]. Ryggkirurgiska kliniken i Strängnäs läggs ned (2020). Available at: https://sverigesradio.se/artikel/7474268 (Accessed 11, 2024).

[41] Martin BI Lurie JD Farrokhi FR McGuire KJ Mirza SK. Early effects of Medicare's bundled payment for care improvement program for lumbar fusion. Spine. (2018) 43:705–11. doi: 10.1097/BRS.0000000000002404, PMID: 28885288 PMC5839918

[42] Bronson WH Kingery MT Hutzler L Karia R Errico T Bosco J. Lack of cost Savings for Lumbar Spine Fusions after Bundled Payments for care improvement initiative: a consequence of increased case complexity. Spine. (2019) 44:298–304. doi: 10.1097/BRS.0000000000002812, PMID: 30045344

[43] Jubelt LE Goldfeld KS Blecker SB Chung WY Bendo JA Bosco JA. Early lessons on bundled payment at an Academic Medical Center. J Am Acad Orthop Surg. (2017) 25:654–63. doi: 10.5435/JAAOS-D-16-00626, PMID: 28837458 PMC6046256

[44] Roland M Guthrie B. Quality and outcomes framework: what have we learnt? BMJ. (2016) 354:i4060. doi: 10.1136/bmj.i4060 27492602 PMC4975019

[45] Flodgren G Eccles MP Shepperd S Scott A Parmelli E Beyer FR. An overview of reviews evaluating the effectiveness of financial incentives in changing healthcare professional behaviours and patient outcomes. Cochrane Database Syst Rev. (2011) 2011:Cd009255. doi: 10.1002/14651858.CD008608.pub2 21735443 PMC4204491

[46] Wilson M Guta A Waddell K Lavis J Reid R Evans C. The impacts of accountable care organizations on patient experience, health outcomes and costs: a rapid review. J Health Serv Res Policy. (2020) 25:130–8. doi: 10.1177/1355819620913141, PMID: 32321282

[47] Wagenschieber E Blunck D. Impact of reimbursement systems on patient care – a systematic review of systematic reviews. Health Econ Rev. (2024) 14:22. doi: 10.1186/s13561-024-00487-6, PMID: 38492098 PMC10944612

[48] Iderberg H Willers C Borgström F Hedlund R Hägg O Möller H. Predicting clinical outcome and length of sick leave after surgery for lumbar spinal stenosis in Sweden: a multi-register evaluation. Eur Spine J. (2019) 28:1423–32. doi: 10.1007/s00586-018-5842-3 30511244

[49] Timpka T Nyce JM Amer-Wåhlin I. Value-based reimbursement in collectively financed healthcare requires monitoring of socioeconomic patient data to maintain equality in service provision. J Gen Intern Med. (2018) 33:2240–3. doi: 10.1007/s11606-018-4661-x, PMID: 30206793 PMC6258603

[50] Chou R Baisden J Carragee EJ Resnick DK Shaffer WO Loeser JD. Surgery for low back pain: a review of the evidence for an American pain society clinical practice guideline. Spine. (2009) 34:1094–109. doi: 10.1097/BRS.0b013e3181a105fc 19363455

[51] Sullivan R Jarvis LD O'Gara T Langfitt M Emory C. Bundled payments in total joint arthroplasty and spine surgery. Curr Rev Musculoskelet Med. (2017) 10:218–23. doi: 10.1007/s12178-017-9405-8, PMID: 28364146 PMC5435636

[52] Kazberouk A McGuire K Landon BE. A survey of innovative reimbursement models in spine care. Spine. (2016) 41:344–52. doi: 10.1097/BRS.0000000000001212, PMID: 26555826

[53] Song Z Safran DG Landon BE Landrum MB He Y Mechanic RE. The “alternative quality contract,” based on a global budget, lowered medical spending and improved quality. Health Aff (Millwood). (2012) 31:1885–94. doi: 10.1377/hlthaff.2012.0327 22786651 PMC3548447

[54] Walshe K. Pseudoinnovation: the development and spread of healthcare quality improvement methodologies. Int J Qual Health Care. (2009) 21:153–9. doi: 10.1093/intqhc/mzp012, PMID: 19383716

[55] Mullen KJ Frank RG Rosenthal MB. Can you get what you pay for? Pay-for-performance and the quality of healthcare providers. Rand J Econ. (2010) 41:64–91. doi: 10.1111/j.1756-2171.2009.00090.x, PMID: 21667575

[56] Busse R Geissler A Aaviksoo A Cots F Hakkinen U Kobel C. Diagnosis related groups in Europe: moving towards transparency, efficiency, and quality in hospitals? BMJ. (2013) 346:f3197. doi: 10.1136/bmj.f3197 23747967

[57] Reindersma T Fabbricotti I Ahaus K Sulz S. Integrated payment, fragmented realities? A discourse analysis of integrated payment in the Netherlands. Int J Environ Res Public Health. (2022) 19:8831. doi: 10.3390/ijerph19148831 35886684 PMC9318584

[58] Mascha EJ Sessler DI. Segmented Regression and Difference-in-Difference Methods: Assessing the Impact of Systemic Changes in Health Care Anesth Analg. (2019) 129:618–33. doi: 10.1213/ANE.0000000000004153 31008746

