# Supplementary material for: A cost-reducing reimbursement programme? Effects of value-based reimbursement on healthcare costs
Source: Front Public Health. 2024 Dec 11;12:1326067. doi: 10.3389/fpubh.2024.1326067 (PMC11668751; doi:10.3389/fpubh.2024.1326067)
Supplement: Supplementary file 1 [file Table_1.docx]

Supplementary Material

**Table S1.** Included categories in the study for degenerative lumbar surgery based on diagnosis and surgical procedure.

| Category | Diagnosis | Surgical procedure |
| --- | --- | --- |
| A | Disc herniation | Discectomy |
| B1 | Spinal stenosis | Decompression |
| B2 | Spinal stenosis | Fusion |
| C | Segmental dysfunction | Fusion |
| D | Spondylolisthesis | Fusion |
