# Supplementary material for: A cost-reducing reimbursement programme? Effects of value-based reimbursement on healthcare costs
Source: Front Public Health. 2024 Dec 11;12:1326067. doi: 10.3389/fpubh.2024.1326067 (PMC11668751; doi:10.3389/fpubh.2024.1326067)
Supplement: Supplementary file 2 [file Table_2.docx]

Supplementary Material 2

**Sensitivity analysis**

The segmented regression analysis for the period from July 2011 to December 2015 shows a negative pre-intervention trend that is strengthened after the introduction of VBRP, both trends are however non-significant for both total episode cost and mean episode costs. After the introduction of VBRP, there is an immediate increase in total costs, totalling € 220 543 per month, though this change is not statistically significant (p=0.143). Meanwhile, the mean cost decreased by €162 per month, which is also not significant (p=0.7405). The parameter adjusting for the month of July show significantly lower cost for Region Stockholm, with a reduction of €1,125,450 (p <.0001) in total costs and €1,377 (p= 0.0019) in mean costs. Figure 3 illustrates the estimated value of the regression analysis alongside the monthly observation.

**Table S2** Parameter estimates predicting the total and mean episode cost of surgically treated patients, July 2011- December 2015 (shorter time frame).

|  |  | **(A) Total episode cost**  **per month (€)** | | |  | **(B) Mean episode cost**  **(€)** | | |
| --- | --- | --- | --- | --- | --- | --- | --- | --- |
| **Parameter** |  | **Estimate** | **SE** | **p-value** |  | **Estimate** | **SE** | **p-value** |
| **Intercept (July 2011)** |  | 1 262 576 | 527 111 | 0.0205 |  | 9 978 | 1 729 | <.0001 |
| **Time** |  | - -1 269 | 6 716 | 0.8509 |  | -5 | 22 | 0.8312 |
| **VBRP (Oct 2013)** |  | 220 534 | 148 135 | 0.1430 |  | -162 | 486 | 0.7405 |
| **Time after** |  | -6 671 | 9 507 | 0.4862 |  | -41 | 31 | 0.1962 |
| **July** |  | -1 125 450 | 128 003 | <.0001 |  | -1377 | 420 | 0.0019 |


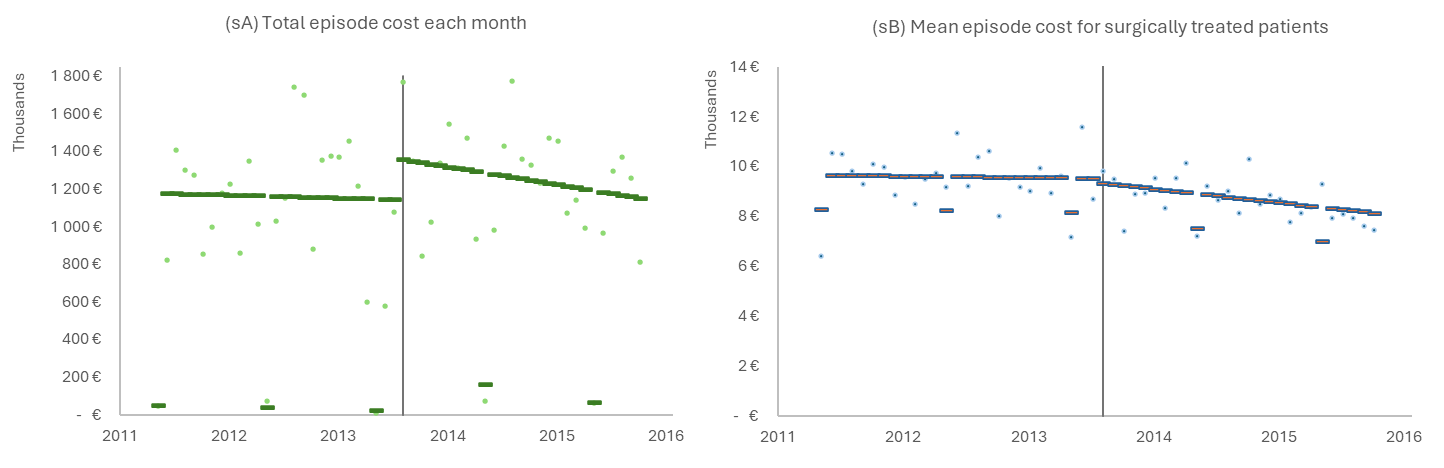


**Figure S1** The total (A) and mean (B) episode cost for surgically treated patients, 2011-2015.
Legend: The vertical line indicates the introduction of the STHLM-VBRP at the end of 2013
